# Dr. Jacob Walter Eleeza Karr Davis

**Published:** 1914-02

**Authors:** 


Dr. Jacob Walter Eleeza Karr Davis, Buffalo 1887, a practitioner of Omaha for 26 years, died December 4, suddenly, in a street car, of heart disease, aged 64.

